# Factors affecting road safety compliance in a low- and middle-income country: An observational study from Lebanon

**DOI:** 10.1371/journal.pgph.0000154

**Published:** 2022-03-28

**Authors:** Samar Al-Hajj, Majed El Hechi, Mohamad Chahrour, Sarah H. Farran, Haytham Kaafarani

**Affiliations:** 1 Epidemiology and Population Health Department, Faculty of Health Sciences, American University of Beirut, Beirut, Lebanon; 2 Division of Trauma, Emergency Surgery and Surgical Critical Care, Massachusetts General Hospital, Boston, Massachusetts, United States of America; New Mexico State University, UNITED STATES

## Abstract

Road traffic injury is a major public health problem in Lebanon. This study aims to assess compliance with safety measures including seatbelt and helmet use in adults and children, and the prevalence of distracted driving among road users across Lebanon different governorates. It further aims to investigate predictors of compliance with seatbelt and helmet use. A cross-sectional observational field study was conducted at multiple governorates in Lebanon. Data collected included information on vehicles, road users and passengers. Univariate and multivariable logistic regression analyses were performed to identify trends in compliance with safety measures and distracted driving, and predictors of compliance. A total of 13,790 road users were observed. The rate of seatbelt and helmet use were 37.4% and 38.9%, respectively, among adults. Distracted behavior was present in 23.7% of car drivers and 22.8% of motorcyles adult riders. Compliance with seatbelt use was lower outside the capital city Beirut [OR = 5.236 (4.566–6.004), *P <0*.*001*], in males [OR = 1.688 (1.52–1.874), *P <0*.*001*], in drivers of taxi/vans [OR = 1.929 (1.71–2.175), *P <0*.*001*] or trucks [OR = 3.014 (2.434–3.732), *P <0*.*001*], and vehicles of lower price [OR = 3.291 (2.836–3.819), *P <0*.*001*]. Children vehicle passengers were 87.9% while motorcycles pillion riders were 12.1%. The rates for child car restraint and helmet use were 25.8% and 20.1%, respectively. Predictors of failure to use a child restraint system in vehicles were the youngest age group (0–5 years) [OR = 2.06, CI (1.40–3.02), *P<0*.*001*], sitting in the back seat [OR = 1.56, CI (1.09–2.23), *P<0*.*001*], ridding in the afternoon [OR = 1.43, CI (1.05–1.94), *P = 0*.*02*], and being outside Beirut [OR = 2.12, CI (1.41–3.17), *P<0*.*00*]. Public awareness efforts and better enforcement of road safety legislations are needed to increase the alarmingly low rates of compliance with safety measures and safeguard lives on the road.

## Introduction

Road traffic injury (RTI) represents a global pandemic causing over 50 million disabilities and claiming approximately 1.35 million deaths worldwide every year [1–3]. For children and youth aged 5 to 29, it is the leading cause of death, surpassing the mortality threat imposed by all major diseases combined [1, 2]. Failure to comply with road safety measures, coupled with distracted and risky behavior drivers, substantially increase the risk, severity and fatalities associated with RTIs [4–7].

Appropriate utilization of seatbelts and helmets has shown to decrease RTI-related morbidity and mortality [4, 8, 9]. Seatbelts prevent occupants from striking the vehicle interior and from being ejected in high impact collisions [10–12]. Helmet use by motorcyclists has shown to play a major role in preventing fatal head injuries [8, 13]. In children, using restraint systems in vehicles and wearing proper have been associated with a major reduction in child RTI severity and fatalities [14–17]. Compliance with safety measures overall was associated with up to 60% reduction in the rate of passengers’ severe and fatal injuries [4].

Abiding with road safety measures remains a critical concern. Only 9% of the world population has been reported to abide with recommendations for child restraint systems, rendering RTI-related injruies, disabilities and fatalities a global health problem [18]. As with other age groups, the majority of road injuries occur in low and middle income countries (LMICs) [1], including those in the Eastern Mediterranean Region (EMR) [19, 20]. In fact, the EMR suffers from a sizeable and disproportionate child road injury burden, ranking second globally in child mortality [21]. EMR countries have low compliance with seatbelt use and consequently high rates of RTI-related severe head injuries [10, 11, 13]. In Lebanon, data on road injuries are scarce and underreported. The most recent estimates indicated an RTI-related mortality rate of 22.3 per 100,000 population [22–24], and a mortality from car crashes specifically of 8% [25]. Lebanon’s absence of public transportation and limited enforcement of road regulations strongly contribute to the observed high RTI rates [1, 26–28]. The Lebanese Road Safety Law 243 drafted in 1967, was reviewed in 2014 to mandated the use of seatbelts and helmets and constrained the use of cellphones while driving [26]. Despite sub-optimal enforcement, the enactment of the revised law in 2015 led to a slight though noticeable decrease in the number of RTI in the country [29].

Despite the severity of road injuries problem in Lebanon, few studies of road user’s safety compliance have been conducted in Lebanon with limited assessment of road safety patterns and predictor factors for non-compliance [22, 23, 30–32]. This study aims to assess road safety practices including seatbelt and helmet use, child restraint system use, and distracted driving among vehicle passengers and motorcycle riders in different Lebanese governorates. The generated knowledge will provide evidence to road safety advocates and law enforcement agencies to inform tailored road safety awareness strategies, regulations, and policies.

## Methods

This is a cross-sectional observational study of safety practices among road users (including drivers and passengers of moving vehicles, riders of motorcycles and bicycles) and child restraint compliance. An roadside observational design was adopted to enhance the investigation of users’ road safety compliance in their setting without interference from the observer. Compared to self-reporting surveys, hospital chart reviews and police reports [33–36], direct roadside observation is considered a reliable method to accurately assess the actual rate of safety compliance. It limits biases introduced by improper documentation of police road violations, and individuals overestimated self-reported compliance to avoid legal liability or social desirability. Direct roadside observational studies offers the advantage of recording road users’ practices within their physical, cultural and social environment without interfering or influencing individuals’ norms and behaviors, and therefore facilitates accurate descriptive documentation of the phenomenon [11]. Such designs are frequently adopted in road safety research [37–39], especially when monitoring road users behaviors and evaluating interventions [40].

### Study setting

This is a cross-sectional roadside observational study among road users. Lebanon is divided into 8 governorates: Beirut, Mount Lebanon, Bekaa, Akkar, Baalbek, Nabatiyeh, North, South (S1 Fig). A stratified multistage sampling strategy was adopted to randomly select one observation site from each strata (governorate) to accommodate for the various socio-economic status, income and education level in different governorate. First, each governorate was delineated as a stratum and included in the study. Next, observation sites were randomly selected from each stratum. These sites were mainly intersections with the following criteria: 1) controlled intersection (with traffic lights, roundabouts or stop signs), 2) major intersection (high density of vehicle traffic, traffic jams where travelling drivers naturally slow down or stop), and 3) lit intersection (to permit night observation). Intersections were identified from a list of the country’s road networks and segmented by governorate.

### Data collection

Roadside observations were conducted over a one-week period from April 27 to May 3, 2019, on weekdays during mornings (7–9 am) and afternoons (3–5 pm) rush hours and during weekends busy hours (i.e. Saturday 7–9 pm and Sunday 4–6 pm). The study period was deemed appropriate based on the methodology adopted in previously published similar studies [33, 37, 41–43].

Eight data collectors were recruited and formally trained then assigned one of the designated observational sites. The training included review of data variables, practice of strategies and effective field observation techniques to ensure fast and accurate data collection. Observers were recruited from each governorate. They were familiar with local roads and traffic flows and advised on the selection of the appropriate observational sites. Observers were stationed in close proximity to moving vehicles and motorcycles for clear observation and accurate estimation of road users’ age and position in the vehicle. For increased accuracy, observers captured data from the closest traffic lane. Lit intersections were selected to permit accurate observations at night.

The study sampling population consists of road users traveling through the selected observation sites during the study period, including drivers of vehicles, passengers (front seat or back seat if a child is onboard) and riders of motorcycles. All traveling road users observed by the data collectors were captured into the study sample. Collected data included vehicle information such as classification (Car, Taxi/Van, Truck, Motorcycle), estimated price (low, medium, high), road users’ gender, estimated age (18–30, 31–45, 46–60, 61+**)**, occupant position (driver, front passenger, back passenger), safety measures used (i.e. seatbelt, helmet, and child restraint system i.e. car seat/booster seat), unsafe behavior (using phones, eating, carrying products on motorcycle while riding). For passengers under 18, data collected included child position in the vehicle/motorcycle and the estimated child age stratified into oldest 12–17, middle 6–11, and youngest child 0–5, in accordance to measurements used in similar observational studies [44–47].

Observed data were collected on electronic data collection forms for accurate and fast capturing (S1 Data). Observations were conducted simultaneously by data collectors at all selected sites to increase inter-observer reliability and data consistency, and accuracy [48]. The data collection process was successfully pilot tested prior to the onset of the roadside observations to ensure data validity. The pilot study served to validate the data collection form, validate age estimation, and refine and clarify definitions. For instance, distracted drivers is defined as drivers being preoccupied with other activities in addition to driving including talking over the phone, texting or looking at their phone, eating/drinking, carrying products while riding a motorcycle [49]. Pilot testing the form also served to achieve inter-observer agreement on the age estimation. Age estimation was adopted in accordance to measurement used in similar observational studies [44–47]. Additional trial observations and training for data collectors were conducted to reduce observer effects.

Informed consent was waived as the study involved no more than minimal risk to participants, and more importantly due to the lack of practicality in soliciting informed consents from individuals observed in a public place. No identification data were collected concerning vehicle registered plate number or any other road user personal identification information. The study was reviewed and approved by the Institutional Review Board at the American University of Beirut.

### Data analysis

The sample size was 13,790 observations, sampled randomly from the 8 governorates (S1 Fig). Similar to existing road observation studies [12, 13, 50, 51], this sample size represents a reasonable indicator of the rate of safety compliance among the Lebanese population. The study sample was stratified by age (children 0–17 and adults road users 18 and above). Descriptive statistical analyses were used to quantify the current level of road users’ and passengers’ compliance with safety measures and distracted driving behaviors. Chi-squared tests were performed to compare categorical data. Variables that were significant on bivariate analysis at a p-value of 0.05 were included in the multivariate model. Logistic regressions were conducted to assess the vehicle and road user related variables that predict failure of compliance with safety measures, specifically seatbelt and helmet use, and predictors in the regression model were considered significant at a p-value of 0.05. STATA v.15 (Stata Corp LP, College Station, TX) was used the statistical analysis.

## Results

Of the total 13,790 roadside observations collected, 12,642 were adults and 1,148 were children. There was an even geographic distribution of observations, with most occurring during weekdays (85.7% for adults and 86.5% for children) and in the afternoon (51.4% and 52.4%, respectively).

### Adult road users

Table 1 shows the distribution of seatbelt use across adult road users and passengers under 18 years that were included in the study. 86.4% of observations in road users were in 4-wheeled vehicles, and 72.5% were male. The age group distribution was 25.2% for age group (18–31), 39.4% for (31–45), 19.9% for (45–60), and 7.2% for above 60 years old. Among adult road users in 4-wheeled vehicles (i.e. cars, taxis, and trucks), only 37.4% (N = 4087) were using seatbelts. For road users in the driver seat, seatbelt use rate was 39.7%. Seatbelt use was less than 50.0% across all adult age groups, with the highest compliance among the 31–45 and 45–60 age group (39.5% and 40.3% respectively). There were significantly more females using seatbelts than males (46.9% versus 33.5%). Geographically, seatbelt compliance was the highest in the capital city Beirut (64.1%), and the lowest in the northern city of Tripoli (15.6%). Seatbelt compliance was the highest in the evening hours (38.3%), with no difference between weekdays and weekends (Table 1).

**Table 1:** Seatbelt use in car/van/truck drivers as stratified across different demographics for adult road users and child passengers.

Road Users above 18				Passengers Under 18		
	No Seatbelt	Seatbelt	P-value	No Seatbelt	Seatbelt	P-value
	N (%)	N (%)		N (%)	N (%)	
	6829 (62.6)	4087 (37.4)		749 (74.2%)	260 (25.8%)	
**Age**			**<0.001**			**<0.001**
0–5*	-	-		254 (82.7)	53 (17.3)	
6–11**	-	-		274 (73.7)	98 (26.3)	
12–17***	-	-		221 (67.0)	109 (33.0)	
18–30	1800 (68.4)	830 (31.6)		-	-	
31–45	2923 (60.5)	1907 (39.5)		-	-	
45–60	1495 (59.7)	1011 (40.3)		-	-	
61+	611 (64.3)	339 (35.7)				
**Gender**			**<0.001**			**0.730**
Female	1701 (53.1)	1501 (46.9)		264 (75.6)	85 (24.4)	
Male	5118 (66.5)	2573 (33.5)		441 (73.6)	158 (26.4)	
Unknown	10 (43.5)	13 (56.5)		44 (72.1)	17 (27.9)	
**Vehicle Type**			**<0.001**			**0.973**
Car	4327 (57.5)	3200 (42.5)		53 (17.3)	254 (82.7)	
Taxi/Van	1923 (71.8)	755 (28.2)		98 (26.3)	274 (73.7)	
Truck	579 (81.4)	132 (18.6)		109 (33.0)	221 (67.0)	
**Price**			**<0.001**			**<0.001**
High	1002 (52.6)	902 (47.4)		121 (57.1)	91 (42.9)	
Medium	3587 (59.8)	2412 (40.2)		422 (75.6)	136 (24.4)	
Low	2240 (74.3)	773 (25.7)		206 (86.2)	33 (13.8)	
**Position**			**<0.001**			**<0.001**
Passenger Front Seat	1519 (66.8)	755 (33.2)		129 (66.8)	64 (33.2)	
Passenger Back Seat	489 (75.6)	158 (24.4)		248 (77.5)	72 (22.5)	
Driver Seat	4807 (60.3)	3171 (39.7)		105 (58.7)	74 (41.3)	
Child (0–5) in Driver’s Lap	-	-		14 (100.0)	0 (0.0)	
Car Seat	-	-		0 (0.0)	44 (100.0)	
In Passenger Lap†	-	-		213 (96.4)	8 (3.6)	
**Behavior**			0.561			
Eating	290 (53.4)	253 (46.6)				
On a call	720 (56.1)	563 (43.9)				
Texting/Browsing	359 (55)	294 (45)				
**Location**			**<0.001**			**<0.001**
Beirut	466 (35.9)	831 (64.1)		89 (62.7)	53 (37.3)	
Bekaa	344 (44.7)	426 (55.3)		111 (49.3)	114 (50.7)	
Jouniyeh	795 (48.2)	854 (51.8)		49 (81.7)	11 (18.3)	
Nabatiyeh	580 (60.9)	373 (39.1)		-	-	
Saida	1015 (76.5)	312 (23.5)		189 (86.3)	30 (13.7)	
Southern Beirut	789 (80.7)	189 (19.3)		-	-	
Tripoli	1813 (84.4)	336 (15.6)		104 (98.1)	2 (1.9)	
Tyre	1027 (57.3)	766 (42.7)		207 (80.5)	50 (19.5)	
**Time of Day**			**0.014**			**0.007**
Morning	2867 (62.9)	1688 (37.1)		307 (69.8)	133 (30.2)	
Evening	3450 (61.7)	2146 (38.3)		404 (77.0)	121 (23.1)	
Night	512 (66.9)	253 (33.1)		38 (86.4)	6 (13.6)	
**Day of Week**			**0.361**			**0.043**
Weekday	5886 (62.7)	3497 (37.3)		640 (73.0)	235 (26.9)	
Weekend	943 (61.5)	590 (38.5)		109 (81.3)	25 (18.7)	

*youngest child,

**middle child,

***oldest child,

^†^in between front seats

Including all collected data on demographics, seating position, vehicle details, location and timing of events, the multivariable logistic regression analysis identified the following predictors of failure to use seatbelts in 4-wheeled vehicles, show in in Table 2: age 31–45 years [OR = 0.9 (0.8–0.9), *P* = 0.034] and age 61 and above [OR = 1.3 (1.1–1.6), *P* = 0.005] compared to 18–30 years, male gender [OR = 1.688 (1.52–1.874), *P <0*.*001*], driving a taxi/van [OR = 1.929 (1.71–2.175), *P <0*.*001*] or truck [OR = 3.014 (2.434–3.732), *P <0*.*001*], lower vehicle price [OR = 3.291 (2.836–3.819), *P <0*.*001*], being a front seat passenger [OR = 1.859 (1.65–2.094), *P <0*.*001*] or back seat passenger [OR = 4.621 (3.714–5.751), *P <0*.*001]*, and driving outside of the capital city [OR = 5.236 (4.566–6.004), *P <0*.*001*].

**Table 2:** Multivariable logistic regression to determine predictor variables for the lack of seatbelts use in cars/vans/trucks among adult road users.

Predictors*	OR (95% CI)	*p* value
**Age**		<0.001
18–30	Reference	-
31–45	0.9 (0.8–0.9)	0.034
45–60	1.0 (0.9–1.2)	0.8
61+	1.3 (1.1–1.6)	0.005
**Gender**		<0.001
Female	Reference	-
Male	1.7 (1.5–1.9)	<0.001
**Vehicle Type**		<0.001
Car	Reference	-
Taxi/Van	1.9 (1.7–2.2)	<0.001
Truck	3.0 (2.4–3.7)	<0.001
**Vehicle Price**		<0.001
High	Reference	-
Medium	1.6 (1.4–1.8)	<0.001
Low	3.3 (2.8–3.8)	<0.001
**Seating Position**		<0.001
Driver Seat	Reference	-
Front Seat, Passenger	1.9 (1.7–2.1)	<0.001
Back Seat, Passenger	4.6 (3.7–5.8)	<0.001
**Region**		<0.001
Beirut	Reference	
Bekaa	1.2 (1.0–1.5)	0.116
Jouniyeh	1.6 (1.4–1.9)	<0.001
Nabatiyeh	4.3 (3.5–5.3)	<0.001
Saida	7.14 (5.9–8.6)	<0.001
Southern Beirut	12.1 (9.8–15.0)	<0.001
Tripoli	16.5 (13.7–19.8)	<0.001
Tyre	3.4 (2.8–4.0)	<0.001
**Time of Day**		0.019
Morning	Reference	-
Afternoon	1.0 (0.9–1.1)	0.754
Night	1.3 (1.1–1.6)	0.009

*Only significant predictors in the model are shown in the table. The following independent variables were included in the model: age, gender, vehicle type, vehicle price, seating position, region and time of observations.

Table 3 shows the distribution of distracted behavior across adult road users, according to baseline demographics. Among 4-wheeled vehicle drivers, the rate of distracted behavior was 23.7% (N = 1,892). The most common type of distracted behavior was phone use, at 19.3% (13.8% phone on the ear and 5.5% texting/browsing), followed by eating (4.5%). Distracted behavior was highest in the age group 18–30 (29.6%) and lowest in age group 61 and above. There was a significantly higher proportion of females compared to males (27.5% vs 22.8%) and drivers of ‘high price’ compared to ‘low price’ vehicles (27.6% vs 22.7%) with distracted behavior while driving.

**Table 3:** Distracted behavior in car/van/truck drivers as stratified across different demographics.

	No Distracted Behavior	Distracted Behavior	P-value
	N (%) = 6086 (100)	N (%) = 1892 (100)	
**Age**			**<0.001**
18–30	1182 (70.4)	498 (29.6)	
31–45	2802 (78.2)	783 (21.8)	
45–60	1493 (77.0)	447 (23.0)	
61+	609 (78.8)	164 (21.2)	
**Gender**			**<0.001**
Female	1239 (72.5)	469 (27.5)	
Male	4827 (77.2)	1422 (22.8)	
Unknown	20 (95.2)	1 (4.8)	
**Price**			**0.001**
High	1022 (72.4)	389 (27.6)	
Medium	3278 (77.0)	980 (23.0)	
Low	1786 (77.3)	523 (22.7)	
**Location**			**<0.001**
Beirut	595 (65.6)	312 (34.4)	
Bekaa	139 (38.6)	221 (61.4)	
Jouniyeh	945 (80.8)	225 (19.2)	
Nabatiyeh	610 (95.2)	31 (4.8)	
Saida	766 (87.3)	111 (12.7)	
Southern Beirut	492 (66.5)	248 (33.5)	
Tripoli	1447 (85.7)	242 (14.3)	
Tyre	1092 (68.5)	502 (31.5)	
**Time of Day**			**<0.001**
Morning	2797 (81.6)	630 (18.4)	
Evening	2922 (74.0)	1025 (26.0)	
Night	367 (60.8)	237 (39.2)	
**Day of Week**			**<0.001**
Weekday	5355 (78.3)	1485 (21.7)	
Weekend	731 (64.2)	407 (35.8)	

Table 4 displays the distribution of hemlet use stratified by different characeristics in adult motorcycle users and child passengers. 38.9% (N = 672) of adult motorcycle users wore helmets. The highest compliance with buckled helmets was observed among those aged 31–45 and 45–60, with a prevalence rate of 48.8% and 47.1% respectively. There was no difference in helmet use between males and females (*P* = 0.655). Most helmet users were in the rider seat (40.3%). No differences in distracted behavior were observed between helmet users and non-users. Geographically, helmet compliance was the highest in Jouniyeh (65.9%), and the lowest in Tripoli (2.6%). There was no difference in helmet use at different times during the day, nor between weekdays or weekends (*P* = 0.070 and *P* = 0.115, respectively).

**Table 4:** Helmet use on motorcycles as stratified across different demographics.

	Road Users Above 18			Passengers Under 18		
	No Helmet	Helmet	P-value	No Helmet	Helmet	P-value
	N (%) =	N (%) =		N (%)	N (%)	
	1054 (100)	672 (100)		111 (79.9)	28 (20.1)	
**Age**			**<0.001**			**0.490**
0–5*	-	-		53 (77.9)	15 (22.1%)	
6–11**	-	-		44 (84.6)	8 (15.4%)	
12–17***	-	-		14 (73.7)	5 (26.3%)	
18–30	596 (70.7)	247 (29.3)		-	-	
31–45	307 (51.2)	293 (48.8)		-	-	
45–60	126 (52.9)	112 (47.1)		-	-	
61+	25 (55.6)	20 (44.4)		-	-	
**Gender**			**0.655**			**0.190**
Female	77 (59.2)	53 (40.8)		17 (68.0)	8 (32.0)	
Male	977 (61.2)	619 (38.8)		94 (82.4)	20 (17.5)	
**Price**			**0.001**			
High	2 (28.6)	5 (71.4)		**-**	**-**	
Medium	86 (49.7)	87 (50.3)		**-**	**-**	
Low	966 (62.5)	580 (37.5)		**-**	**-**	
**Position**			**0.024**			**0.025**
Rider Seat	881 (59.7)	595 (40.3)		20 (76.9)	6 (23.1)	
Passenger Back Seat	132 (66.3)	67 (33.7)		49 (90.7)	5 (9.3)	
Passenger Front Seat	32 (76.2)	10 (23.8)		42 (71.2)	17 (28.8)	
**Behavior**			**0.17**	**N/A**	**N/A**	
Carrying Products	189 (55.1)	154 (44.9)				
Eating	5 (55.6)	4 (44.4)				
Using the Phone	27 (71.1)	11 (28.9)				
**Location**			**<0.001**			**<0.001**
Beirut	105 (36.7)	181 (63.3)		7 (87.5)	1 (12.5)	
Bekaa	67 (37.6)	111 (62.4)		16 (44.4)	20 (55.5)	
Jouniyeh	28 (34.1)	54 (65.9)		0 (0.0)	4 (100.0)	
Nabatiyeh	76 (86.4)	12 (13.6)		**-**	**-**	
Saida	53 (48.2)	57 (51.8)		5 (71.4)	2 (28.6)	
Southern Beirut	103 (61.3)	65 (38.7)		**-**	**-**	
Tripoli	371 (97.4)	10 (2.6)		28 (100.0)	0 (0.0)	
Tyre	251 (58)	182 (42)		55 (98.2)	1 (1.8)	
**Time of Day**			**0.07**			**0.410**
Morning	431 (63.5)	248 (36.5)		45 (84.9)	8 (15.1)	
Evening	523 (58.5)	371 (41.5)		58 (75.3)	19 (24.7)	
Night	100 (65.4)	53 (34.6)		8 (88.9)	1 (11.1)	
**Day of Week**			**0.115**			**0.570**
Weekday	870 (60.2)	574 (39.8)		93 (78.8)	25 (21.2)	
Weekend	184 (65.2)	98 (34.8)		18 (85.7)	3 (14.3)	

*youngest child,

**middle child,

***oldest child

As evident in Table 5, multivariable logistic regression analysis adjusting for age, seating position, motorcycle price, and region, identified predictors of failure to use helmets (either buckled or unbuckled) on motorcycles to be ages 31–45 [OR = 0.7 (0.6–0.9), *P* <0.011], 45–60 [OR = 0.8 (0.6–1.3), *P* = 0.414] and 61+ [OR = 1.4 (0.7–2.9), *P* = 0.33], lower motorcycle price [OR = 23.148 (2.468–217.096), *P = 0*.*006*] and driving in the southern and northern regions (i.e. Nabatiyeh, Saida, Southern Beirut suburb, Tripoli, or Tyre) as compared to the capital city, Beirut [OR = 4.826 (3.623–6.430), *P<0*.*001*] (Table 5). Compared to motorcycle passengers (pillions), motorcycle drivers were more likely to use helmets [OR = 0.367 (0.263–0.513), *P<0*.*001*].

**Table 5:** Multivariable logistic regression to determine the predictor variables for the lack of helmets use among adult road users.

Predictors*	OR (95% CI)	p value
**Age**		0.015
18–30		
31–45	0.7 (0.6–0.9)	0.011
45–60	0.8 (0.6–1.3)	0.414
61+	1.4 (0.7–2.9)	0.33
**Motorcycle Price**		0.001
High	Reference	
Medium	13.1 (1.4–125.7)	-
Low	23.1 (2.5–217.1)	0.026
		0.006
**Seating Position**		<0.001
Non-Rider Seat	Reference	-
Rider Seat	0.4 (0.3–0.5)	<0.001
**Region**		<0.001
Beirut	Reference	
Bekaa	1.1 (0.7–1.9)	0.65
Jouniyeh	1.1 (0.6–2.0)	0.704
Nabatiyeh	11.1 (5.7–21.5)	<0.001
Saida	2.0 (1.3–3.1)	0.003
Southern Beirut	2.7 (1.8–4.0)	<0.001
Tripoli	88.4 (43.3–180.5)	<0.001
Tyre	2.6 (1.8–3.7)	<0.001

*Significant predictors in the model are shown in the table. The following independent variables were included in the model: age, vehicle price, seating position, and region of observation.

Table 6 displays the distribution of distracted behavior in adult users, stratified by different characteristics. Among motorcycle riders, the rate of all distracted behavior was 22.8% (N = 336). The highest distracted behavior was carrying products (19.6%) followed by phone use (2.5%). Distracted behavior was highest in the age group 18–30 and lowest in age group 45–60. Riders of ‘low price’ compared to high price vehicles (24.0% vs 13.0%) were more likely to ride with distracted behavior.

**Table 6:** Distracted behavior in adult motorcycle rider as stratified across different demographics.

	No Distracted Behavior	Distracted Behavior	P-value
	N (%) = 1140 (100)	N (%) = 336 (100)	
**Age**			**0.001**
18–30	549 (74.3)	190 (25.7)	
31–45	382 (77.6)	110 (22.4)	
45–60	175 (87.5)	25 (12.5)	
61+	34 (75.6)	11 (24.4)	
**Gender**			0.058
Female	30 (90.9)	3 (9.1)	
Male	1110 (76.9)	333 (23.1)	
**Price**			**0.004**
High	7 (100.0)	0 (0.0)	
Medium	127 (87.0)	19 (13.0)	
Low	1006 (76.0)	317 (24.0)	
**Location**			**<0.001**
Beirut	159 (65.7)	83 (34.3)	
Bekaa	87 (75.7)	28 (24.3)	
Jouniyeh	65 (87.8)	9 (12.2)	
Nabatiyeh	74 (98.7)	1 (1.3)	
Saida	91 (84.3)	17 (15.7)	
Southern Beirut	65 (43.9)	83 (56.1)	
Tripoli	309 (83.7)	60 (16.3)	
Tyre	290 (84.1)	55 (15.9)	
**Time of Day**			**0.038**
Morning	473 (80.6)	114 (19.4)	
Evening	563 (74.7)	191 (25.3)	
Night	104 (77.0)	31 (23.0)	
**Day of Week**			**0.016**
Weekday	969 (78.4)	267 (21.6)	
Weekend	171 (71.3)	69 (28.7)	

### Children as vehicle and motorcycle passengers

87.9% of observations were children in 4-wheeled vehicles (i.e. Cars, Taxis, and Truck) and 12.1% were on motorcycles. Almost 70.0% of children were 11 years old or younger, and 62.1% were male. Among children in 4-wheeled vehicles, only 25.8% (n = 260) adopted a form of restraint systems. Safety compliance (e.g. seatbelt, child restraint) was less than 35.0% across all three age groups, ranging between 17.3% for those younger than 5 years old, and 33.0% among those aged 12–17 years. Males and females were equally likely to have low compliance with restraint measures (26.4% and 24.4% respectively). Young children were observed seated on drivers’ lap (n = 14) and on front seat passenger’s lap (n = 221). In addition to the observed under age adolescent drivers (n = 179), most children seatbelt use was documented in the front passenger seat (33.2%). Geographically, child safety compliance was highest in the Bekaa (50.7%), and lowest in Tripoli (1.9%). Safety compliance was highest in the morning hours (30.2%), and on weekdays as opposed to weekends (26.9% vs 18.7%) (Table 1).

On multivariable logistic regression, predictors of failure to use a child restraint system in 4-wheeled vehicles included younger age groups children (OR = 2.06, CI (1.40–3.02), (*P <0*.*001)*, not sitting in the front seat (OR = 1.56, CI (1.09–2.23), (*P <0*.*001)*, being in a vehicle in the afternoon (OR = 1.43, CI (1.05–1.94), (*P = 0*.*02)* as compared to morning hours, and being in a vehicle outside of the capital city, Beirut (OR = 2.12, CI (1.41–3.17), (*P <0*.*001)* (Table 7).

**Table 7:** Multivariable logistic regression to determine the predictor variables for the lack of seatbelts use for children in cars/vans/trucks.

Predictors*	OR (95% CI)	P-value
**Age**		0.015
12–17 [Large Child]	Reference	
6–11 (Medium Child)	1.4 (1.0–1.9)	0.05
0–5 (Small Child)	2.06 (1.4–3.0)	<0.001
**Seating Position**		<0.001
Passenger Front Seat	Reference	
Not being in the front Seat	1.6 (1.1–2.2)	0.010
**Time of Day**		0.007
Morning	Reference	-
Afternoon	1.4 (1.1–1.9)	0.020
Night	2.4 (0.8–6.9)	0.110
**Region**		<0.001
Beirut	Reference	-
Location outside Beirut	2.12 (1.4–3.2)	<0.001

* Only significant predictors in the model are shown in the table. The following independent variables were included in the model: age, seating position, region and time of observations.

As for children motorcycle pillion passengers, only 20.1% (n = 28) wore helmets. Helmet use was less than 30% across all children age groups, ranging from 15.4% among the age group 6–11 years to 26.3% among the older age group 12–17 years. There was no difference in helmet use between age groups nor between genders. Most helmet uses were documented in children seated in front of the rider (28.8%). Geographically, child helmet use was highest in Jouniyeh (n = 4, 100%) and Bekaa (n = 20, 55.5%), and lowest in Tripoli and Nabatiyeh (0.0%). There was no difference in child helmet use at different times during the day, nor between weekdays or weekends (P = 0.41 and P = 0.57, respectively) (Table 4). Due to the low numbers of observations of children as motorcycle pillion passengers and the rarity of helmet use, multivariable logistic regression could not be performed to determine predictors of helmet use.

## Discussion

To the best of our knowledge, this is the first field study to estimate the rate of use of safety measures use in adults and children road users, and assess the prevalence of distracted driving behavior among road users in Lebanon.

The observed rate of adult seatbelt compliance in Lebanon was alarmingly low (37.4%) compared to rates reported in high-income countries (80.0 to 98.0%) such as the United States, Canada and the UK [52]. Moreover, Lebanese compliance rates remain low in comparison to reported seatbelt use in the Eastern Mediterrenean Region countries including Kuwait (50.0 to 65.0%), Qatar (72.2%), Saudi Arabia (33.0 to 87.0%) and the UAE (61.0%) [10–12, 36, 53]. While a noticeable decline in road injuries was observed in high- and middle-income countries as part of the World Health Organization’s Sustainable Development Goals (SDG), no reduction- and oftentimes an increase- in road deaths and injuries were reported in LMICs [18].

Traffic characteristics and road safety compliance varied between geographic locations, with major discrepancies across governorates. Compliance with road users safety measures was higher in the capital city Beirut, compared to suburbs and remote cities. Road users in rural locations were overall less likely to use seatbelts or helmets compared to urban locations, possibly due to higher public awareness among the urban population and stronger law enforcement. Low socioeconomic status (SES), inferred from estimated vehicle prices, was another predictor of low seatbelt compliance in adults, aligning with existing evidence that indicate the strong association between low SES and low seatbelt use rates [11–13]. The higher compliance rate observed in new model vehicles is possibly linked to the fact that these vehicles have embedded seatbelt alert systems that prompt passengers to use seatbelts, hence increasing compliance [54, 55]. Moreover, many poor families tend to occupy vehicles beyond their capacity, which challenges the appropriate use of seatbelt among occupants. Similarly, helmet use was low among riders of lower priced motorcycles. This aligns with previous findings suggesting that lower SES and high costs of helmets act as barriers to helmet use [56, 57].

In this study, only 25.8% of children were placed in a car child restraint system (i.e. car seat, booster seat or seat belt) and 20.1% wore a helmet on a motorcycle. This remarkable low rate inclines towards LMICs’ reported low child restraint compliance rates that range globally from 99.0% in developed countries to almost 6.1% in developing countries [58–60]. Similar to available evidence from LMICs, our results revealed a high proportion of children under age 12 seated as unrestrained front seat occupants (i.e. children 6–11), or placed on an adult’s lap (i.e. children 0–5) [61]. Compared to the rear-seat position, children seated in front positions, restrained or unrestrained, are at a higher risk of sustaining serious and fatal injuries from collisions [62]. Moving to the rear seat decreases the child’s risk of severe and fatal injury by up to 35.0% [63]. A concerning phenomenon was children’s use of adult seatbelts in many front-seat passenger. Children restrained and fastened with adult seatbelts are four times more likely to sustain severe injuries including head traumas compared to age-appropriate child restraint systems. Children’s bodies are fragile and their small physical statures are vulnerable to high impact collisions [63, 64]. They are prone to experiencing the ‘seatbelt syndrome’, leading to spinal and abdominal injuries during collisions [65, 66].

This study highlighed the low prevalence of helmet use among children motorcycle riders and pillion passengers (20.1%). Motorcycles are utilized by many low SES status families as an affordable mode of transport. Despite motorcyclists’ increased risk of sustaining serious and fatal injuries (approximately 8 folds), many parents fail to comply with the use of helmet or other protective gear for their children [17, 67].

A significant number of underage vehicle drivers and motorcycle riders was recorded. This behavior poses a threat and jeopardizes the safety of children as well as other road users. Despite its documented health risks for drivers and passengers [68–70], unlicensed underage driving is a prevailing norms in Lebanon. The observed rate of distracted behavior (eating or using the phone) while driving was also alarmingly high (23.7%). The rate of cell phone use among car drivers (19.3%) was remarkably higher compared to the rates observed in high income countries (HICs) like the UK, Spain, Canada and the United States with rates ranging between 1.3% and 8.8% [71–75]. The rate of cell phone use was also higher than rates reported in EMR countries such as Qatar (11.5%) and Iran (10%) [76, 77].

Consistent with findings in the literature, this study revealved that distracted behavior was higher in young adult drivers [49, 78, 79]. There is evidence that individuals who engage in distracted behaviors are more impulsive decision-makers with lower levels of executive function, and an inflated perceived ability to multi-task [80–83]. There were significantly more males demonstrating distracted behavior in this cohort compared to females. Previous studies show inconsistent outcomes with no difference, or even a higher association, between females and distratcted driving [78, 84]. Distracted driver behavior was more frequently observed in higher price vehicles, possibly because expensive vehicles, specifically SUVs, might provide drivers with a higher sense of safety, increasing their risk-taking behaviors [76].

Enforcement and imposed sanctions on road safety violations have been previously shown to positively influence the level of safety compliance [85]. For instance, an increase in seatbelt violation fine in the US led to a significant increase in seatbelt use, of 26.0 to 38.0% [85]. The Canadian enforcement model (i.e. combining intensive and sustained enforcement with publicity about the enforcement) has also shown effectiveness. These learned lessons from HICs can be contextualized and tailored to LMICs to enhance road safety laws and legislations, substantially reducing RTIs and victim’s hospitalization stays and the associated financial burden that drains limited healthcare systems resources [24, 86].

This study draws a concise picture of the lack of safety compliance in Lebanon, with relevant implications on safety policies and practices. The provided evidence serves as a key indicator to guide the design of road safety policies and advance public health research and practices, including:

### Enact and enforce road safety policies

In Lebanon, the absence of robustly enforced road safety policies is largely responsible for the observed failure in road safety compliance. For instance, the revised Road Safety Law 243 enacted in 2015 in Lebanon, the lack of helmet use while riding a motorcycle is penalized by a monetary fine and the temporary confiscation of the motorcycle [87]. Nonetheless, this law is rarely enforced and more importantly fails to address the safety of children as motorcycles pillion riders. This study generated knowledge presents relevant data to inform new road safety laws and amend existing ones to align with international standards and best practices, and enhancing road safety, thus meeting SDG’s goals. Moreover, The observed rate of distracted drivers (i.e. cell phone use) is alarming and calls for Lebanese laws to ban the use of cell phone while driving. Banning cell phone use have shown effective in reducing the rate of drivers’ handheld phone usage and consequently road injuries and fatalities [88, 89], and the enactment of such laws is vital in Lebanon.

### Design and development of public health practices and programs

This study provides the evidence that can help to strategically design public health awareness campaigns on the safety benefits of seatbelts and standard helmets use and their impacts on reducing injuries. Education and targeted child road safety interventions should be developed to inform parents about the importance of using a child restraint system to prevent child severe and fatal road injuries. Findings from this study provide preliminary compliance rate that can be adopted to design data-driven road and tailored safety interventions and safety practices programs. Educational campaigns and programs coupled with law enforcement are essential to raise awareness, encourage compliance and enhance safety measures adopted by road users.

This study has some limitations owing to its observational nature [90]. The cross-sectional observational design provided the opportunity to witness behaviors in their natural settings without interference and without influencing road users’ behaviors. However, the study is limited in its ability to make causal relationship or inferences about the various road behavior factors. The study selection bias could be another limitation. Observations were conducted at selected cities in Lebanon’s governorates, which might potentially limit the generalizability of the study findings to all the Lebanese population. Another potential limitation for this observational studies is the presence of confounding bias due to the lack of possible randomization of observed road users or the arbitrary allocation of risk factors to observed drivers and passengers. Information bias could also be another possible limitation of this observational study, referring mainly to the inaccurate evaluation of compliance rate and utilization of assessement measures. Within the context of these observed estimates, the data captured in this study could have underestimated the actual prevalence of road safety lack of compliance. Finally, the study did not investigate the proper use of seatbelts, helmets and children restrain systems, all of which can be equally important in protecting drivers and passengers from severe injuries and deaths.

## Conclusion

This study uncovered the alarming low rates of compliance with seatbelt, helmet and child restraint system use, and the high prevalence of distracted driving among road users in Lebanon. The study also highlighted the need to enforce child road safety, eliminate underage and unlicensed driving and increase child restraint systems, thus promoting child safety and well-being. These results provide an opportunity to design data-driven road safety awareness programs to enhance knowledge, and inform road injury prevention strategies to improve safety practices in road users. Future observational research is needed to understand the pattern of changes in compliance rates following public education and law enactment.

## Supporting information

S1 Fig Map of Lebanon showing the different governorates. Source: https://commons.wikimedia.org/wiki/File:Lebanon_governorates_english.svg.
(TIFF)

S1 Data Collected observational data compiled in an excel sheet. (XLSX)
