# Supplementary figures and images for: Factors affecting road safety compliance in a low- and middle-income country: An observational study from Lebanon
Source: PLOS Glob Public Health. 2022 Mar 28;2(3):e0000154. doi: 10.1371/journal.pgph.0000154 (PMC10021161; doi:10.1371/journal.pgph.0000154)

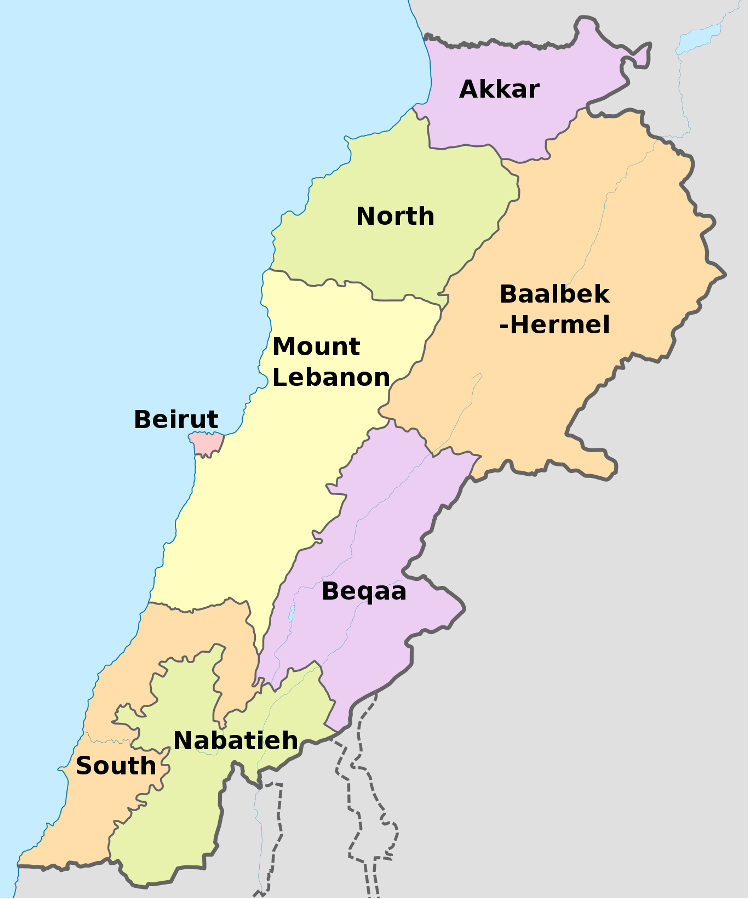

Supplement: S1 Fig — Source: https://commons.wikimedia.org/wiki/File:Lebanon_governorates_english.svg. (TIFF) [file pgph.0000154.s001.tiff]
